# *Macrophominavaccinii* sp. nov. causing blueberry stem blight in China

**DOI:** 10.3897/mycokeys.55.35015

**Published:** 2019-06-19

**Authors:** Lin Zhao, Jing Cai, Wei He, Ying Zhang

**Affiliations:** 1 Beijing Key Laboratory for Forest Pest Control, Beijing Forestry University, Beijing 100083, China Beijing Forestry University Beijing China; 2 Institute of Microbiology, P.O. Box 61, Beijing Forestry University, Beijing 100083, China Beijing Forestry University Beijing China

**Keywords:** *
Vaccinium
*, stem blight, Botryosphaericeae, taxonomy, pathogenicity

## Abstract

Blueberries (*Vaccinium* spp.) have been widely cultivated in China because of their nutritional benefits and economic value. Blueberry stem blight has become one of the most severe diseases influencing blueberry productivity and quality in China. In this study, eight fungal isolates were obtained from twenty stem blight lesions of blueberry collected in Nanping, Fujian province, China. Asexual stage was observed after inducing sporulation, the morphology of which agrees with *Macrophomina* in the black, smooth, hard sclerotia and ellipsoid to obovoid, smooth hyaline conidia with apical sheath. Furthermore, DNA sequences of concatenated ITS, *tef1-α*, *TUB*, and *ACT* loci indicated that these isolates belong to a novel fungal species. The distinguishing morphological characteristics, such as the wider conidia and larger conidiomata pycnidial, also support its new status. Thus a novel fungus, *Macrophominavaccinii*, was described in this study. Pathogenicity tests indicated that *M.vaccinii* could cause stem blight of blueberry.

## Introduction

Blueberries (*Vaccinium* spp.) are popular fruits because of their health benefits health, such as enhancing brain memory and preventing heart disease (Shi and Liu 2009, Popović et al. 2018). Blueberries have been commercially cultivated worldwide, particularly in the USA, Canada and a few European countries (Evans and Ballen 2014). Blueberry cultivation in China started in 1981, and the planted area has reached 31,210 hectares with total production of 114, 905 t in 2017 (Li et al. 2018). Blueberries have been widely cultivated mainly in Guizhou, Shandong and Liaoning province (Xu et al. 2015, Li et al. 2018). Stem blight disease was one of the most prevalent diseases in blueberry cultivation areas in China, which has seriously affected the growth of blueberry plants, fruit quality and productivity (Yu et al. 2012, 2013a, b, Xu et al. 2015, Xu 2016).

A number of fungal species have been reported causing stem blight, dieback or stem canker of blueberries. For instance, *Botryosphaeriadothidea*, *Lasiodiplodiatheobromae*, *Neofusicoccumribis*, and *N.parvum* caused stem blight of highbush or rabbiteyes blueberries in USA (Milholland 1972, Creswell and Milholland 1988, Smith 2004, Wright and Harmon 2009, 2010, Koike et al. 2014). *Macrophominaphaseolina* (Tassi) Goid caused stem blight of highbush blueberries in Serbia (Popović et al. 2018). *Neofusicoccumparvum* caused stem blight and dieback of highbush blueberries in Mexico (Boyzo-Marin et al. 2016). *Diaportheambigua*, *D.australafricana*, *D.neotheicola*, *D.passiflorae*, *Pestalotiopsisclavispora*, *P.neglecta*, and *Truncatellaangustata* caused stem canker and dieback of highbush blueberries in Chile (Espinoza et al. 2008, Elfar et al. 2013), and *Godroniacassandrae* caused stem dieback of highbush blueberry in Norway (Stromeng and Stensvand 2011).

The genus *Macrophomina* was introduced based on *M.phaseolina*, and assigned in the Botryosphaeriaceae (Botryosphaeriales) (Crous et al. 2006, Phillips et al. 2013). Thus far, three species are accommodated within *Macrophomina*, *viz. M.phaseolina*, *M.pseudophaseolina* Crous, Sarr & Ndiaye, and *M.euphorbiicola* A.R. Machado, D.J. Soares & O.L. Pereira (Phillips et al. 2013, Sarr et al. 2014, Machado et al. 2019). *Macrophominaphaseolina* is a soil- or seed-borne polyphagous pathogen, causing charcoal rot disease on about 500 plant species of more than 100 families throughout the world (Su et al. 2001, Babu et al. 2007, Sarr et al. 2014). In Serbia, *M.phaseolina* was reported as a causal agent causing foliage death and brown discoloration of internal vascular stem tissues of highbush blueberry in 2015 (Popović et al. 2018). So far, *M.pseudophaseolina* has been reported causing charcoal rot disease on six plant species, *viz. Abelmoschus esculentus*, *Arachishypogaea*, *Hibiscussabdariffa*, *Vignaunguiculata*, *Gossypiumhirsutum*, *Ricinuscommunis*, and associated with seed decay of *Jatrophacurcas* (Sarr et al. 2014, Machado et al. 2019). *Macrophomiaeuphorbiicola* has only been reported as the causal agent of the charcoal rot on *Ricinuscommunis* and *Jatrophagossypiifolia* (Machado et al. 2019).

In the course of an ongoing survey of biodiversity of fungi causing stem blight of blueberries in China, a new taxon with general characteristics of *Macrophomina* was collected. The aim of this study was to identify the new isolates based on morphological characteristics and multigene phylogenetic analysis, and determine their pathogenicity on the blueberry.

## Materials and methods

### Sample collection, fungal isolation and morphological studies

This study was conducted at the Blueberry Production Garden in the suburb area of Nanping, Fujian province, China. Twenty diseased or dead stems (about 30 cm in length) were collected from blueberry branches in February, 2018. Wood segments (0.5 × 0.5 × 0.2 cm) cut from the diseased lesion boundary or dead tissue were surface sterilized (Pavlic et al. 2004) and incubated on malt extract agar (MEA, 2%) for fungal strains. Petri-dishes were incubated in the dark at 28 °C until fungal colonies were observed. Pure cultures were obtained by hyphal tips from the margin of the suspected *Macrophomina* colonies, which were subcultured on fresh MEA and maintained at 28 °C.

To induce sporulation of conidia, isolates were cultivated on synthetic nutrient-poor agar (SNA) with autoclaved pine needles placed onto the medium, and incubated at 25 °C under near-UV light (mainly 340 nm) (Dou et al. 2017b). Pycnidia produced on the pine needles were morphologically described and characterized following the protocol of Dou et al. (2017a, b). Measurements of conidia, conidiogenous cells and microconidia were made from water mounts. Measurements and digital photographs were made using a Nikon Coolpix 995 digital camera connected to a trinocular Leitz Orthoplan microscope and processed with Adobe Photoshop Elements 10 software. Fungal isolates and specimens were deposited at Beijing Forestry University (BJFU) with duplicates in the China General Microbiological Culture Collection Center (CGMCC) and the Mycological Herbarium of the Institute of Microbiology, Chinese Academy of Sciences (HMAS) (Table 1).

**Table 1.** GenBank accession numbers of isolates included in this study (newly generated sequences are in bold).

Species	Sample number	GenBank accession number			
		ITS	*tef1-a*	*TUB*	*ACT*
* Botryosphaeria dothidea *	CBS 115476	AY236949	AY236898	AY236927	–
	CBS 110302	AY259092	AY573218	EU673106	–
* Macrophomina euphorbiicola *	CMM 4045	KU058928	KU058898	MF457657	MF457654
	CMM 4134	KU058936	KU058906	MF457658	MF457655
	CMM 4145	KU058937	KU058907	MF457659	MF457656
* M. phaseolina *	CBS 162.25	KF531826	KF951996	KF531805	KF951803
	CBS 227.33	KF531825	KF952000	KF531806	KF951807
	CPC 21388	KF951703	KF952074	KF952165	KF951843
	CPC 21392	KF951705	KF952076	KF952167	KF951844
	CPC 21395	KF951706	KF952077	KF952168	KF951846
	CPC 21399	KF951707	KF952078	KF952169	KF951847
	CPC 21443	KF951734	KF952104	KF952194	KF951872
	CPC 21444	KF951735	KF952105	KF952195	KF951873
	CPC 21445	KF951736	KF952106	KF952196	KF951874
* M. pseudophaseolina *	CPC 21394	KF951786	KF952148	KF952228	KF951913
	CPC 21402	KF951789	KF952151	KF952231	KF951916
	CPC 21403	KF951790	KF952152	KF952232	KF951917
	CPC 21417	KF951791	KF952153	KF952233	KF951918
	CPC 21459	KF951794	KF952156	KF952236	KF951921
	CPC 21501	KF951796	KF952158	KF952238	KF951923
	CPC 21524	KF951799	KF952161	KF952240	KF951925
	CPC 21527	KF951801	KF952163	KF952242	KF951927
	CPC 21528	KF951802	KF952164	KF952243	KF951928
* M. vaccinii *	CGMCC 3.19503	**MK687450**	**MK687426**	**MK687434**	**MK687442**
	CGMCC 3.19504	**MK687451**	**MK687427**	**MK687435**	**MK687443**
	CGMCC 3.19505	**MK687452**	**MK687428**	**MK687436**	**MK687444**
	CGMCC 3.19506	**MK687453**	**MK687429**	**MK687437**	**MK687445**
	CGMCC 3.19507	**MK687454**	**MK687430**	**MK687438**	**MK687446**
	CGMCC 3.19508	**MK687455**	**MK687431**	**MK687439**	**MK687447**
	CGMCC 3.19509	**MK687456**	**MK687432**	**MK687440**	**MK687448**
	CGMCC 3.19510	**MK687457**	**MK687433**	**MK687441**	**MK687449**

### DNA extraction, PCR amplification and sequencing

DNA was extracted from mycelia grown on MEA plates with CTAB plant genome DNA fast extraction kit (Aidlab Biotechnologies Co., Ltd, Beijing, China). The internal transcribed spacer of rDNA (ITS) was amplified and sequenced with primers ITS-1 and ITS-4 (White et al. 1990). The translation elongation factor-1α (*tef1-α*) was amplified and sequenced with primers EF1-688F and EF1-1251R (Alves et al. 2008). The β-tubulin gene (*TUB*) was amplified and sequenced with primers Bt2a and Bt2b (Glass and Donaldson 1995). The actin gene (*ACT*) was amplified and sequenced with primers ACT-512F and ACT-2RD (Carbone and Kohn 1999, Sarr et al. 2014). PCR amplification and sequencing followed the protocols of Zhang et al. (2009).

### Sequence alignment and phylogenetic analysis

DNA sequences of concatenated ITS, *tef1-α*, *TUB*, and *ACT* loci were analyzed to investigate the phylogenetic relationships among *Macrophomina* species with DNA sequences available from GenBank (http://www.ncbi.nlm.nih.gov/genbank/), as well as the sequences generated herein (Table 1). A multiple alignment was conducted with MEGA v. 6 (Tamura et al. 2013) and analyses were performed in PAUP V. 4.0b10 (Swofford 2002). Prior to phylogenetic analysis, ambiguous sequences at the start and the end were deleted and gaps manually adjusted to optimize the alignment. Maximum parsimony (MP) was conducted with heuristic searches as implemented in PAUP with the default options method (Zhang et al. 2008). Analyses with gaps treated as missing data were conducted under different parameters of maximum parsimonious criteria as outlined in Zhang et al. (2008). Clade stability was evaluated in a bootstrap analysis with 1,000 replicates, random sequence additions with the maxtrees set to 1,000 and other default parameters as implemented in PAUP. Maximum likelihood (ML) was also conducted using heuristic searches with the default options method as implemented in PAUP. For the ML analysis, best-fit model of nucleotide evolution (HKY+G) was selected by hierarchical likelihood ratio test (hLRT) in MrModeltest 2.3 (Posada and Crandall 2001). A bootstrap analysis with 1,000 replicates was used to test the statistical support of the branches. Trees were viewed in TreeView 1.6.6 (Page 1996). The nucleotide sequences reported in this paper were deposited in GenBank. Trees and alignments were deposited in TreeBase (https://treebase.org/treebase-web/home.html, submission ID: 24410).

### Pathogenicity test

Three isolates of *Macrophominavaccinii* (CGMCC 3.19503, CGMCC 3.19505, and CGMCC 3.19510) obtained in this study were used to conduct a pathogenicity test. The pathogenicity test was performed on 2-year blueberry stems (*cv.* O’Neal) in a humid chamber at 28 °C with semi-shaded conditions. Stems for inoculation were surface sterilized with 75% ethanol for 1 min before making a tangential cut (5 mm in length) on the bark (Espinoza et al. 2009). A 5-mm-diameter MEA medium with mycelial was taken from the 3-day colony, which was placed on to the wounded site, and subsequently covered with parafilm. Three replicates were conducted for each isolate. Noncolonized MEA agar plugs were used as negative controls. Pathogenicity was determined by the length of the necrotic lesion caused by the tested isolates three weeks after inoculation. Fungal isolates were re-isolated from the infected tissue, and morphological characterization and DNA sequence comparisons were conducted to fulfill Koch’s postulates. Mean comparisons were conducted using Tukey’s Honest Significant Difference test (HSD, α = 0.05) in R (Version 3.2.2, R Inc. Auckland, NZL).

## Results

### Phylogeny

**Figure 1.**
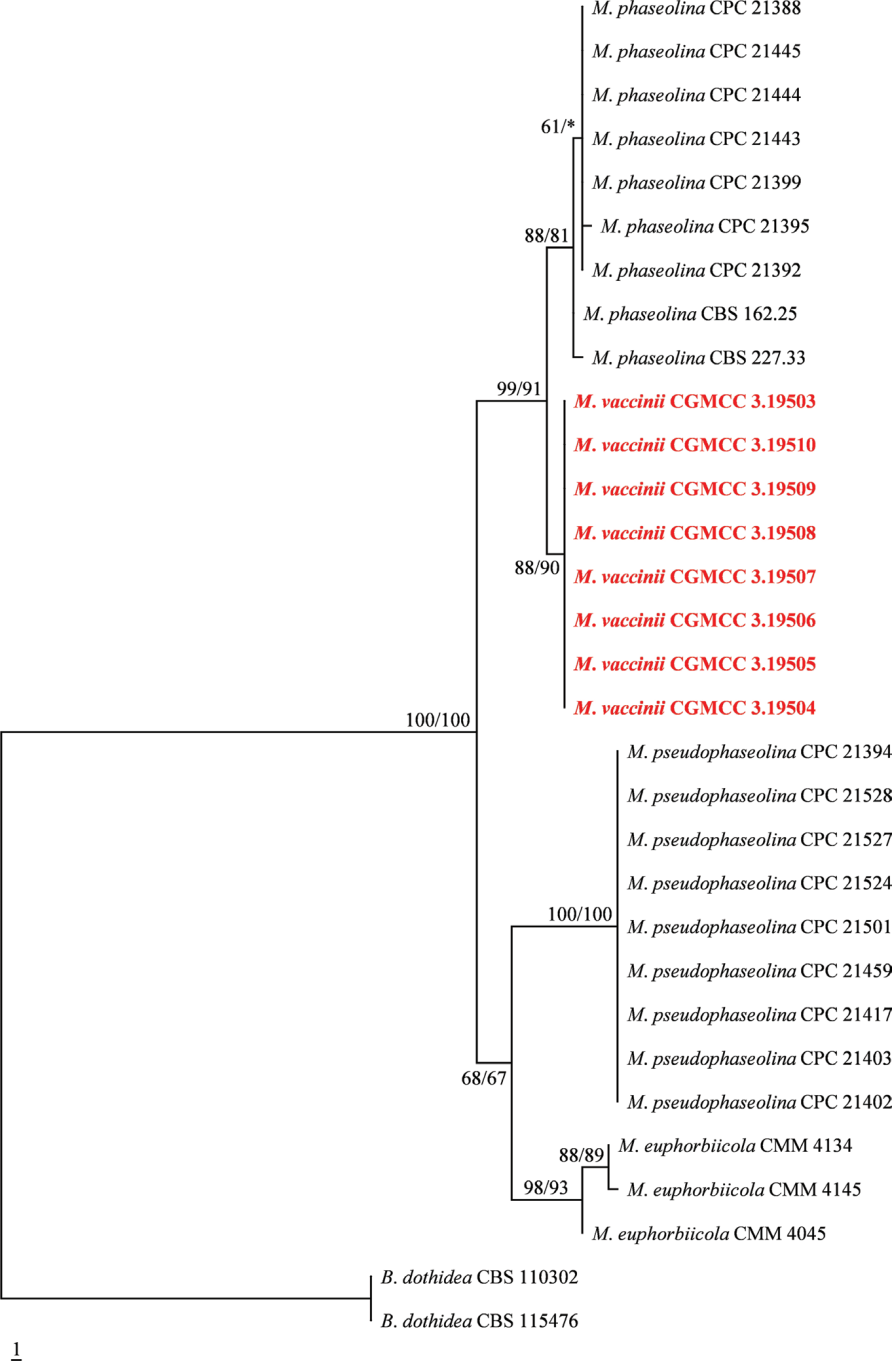
Maximum parsimony tree generated from sequence analysis of the concatenated ITS, *tef1-α*, *TUB* and *ACT* dataset. Designated out group taxa is *B.dothidea*. Maximum parsimony (MP) and maximum likelihood (ML) bootstrap support greater than or equal to 60% are shown above the nodes (* = value less than 60%). The positions of the *Macrophominavaccinii* isolates are indicated in bold and red text.

Phylogenetic analysis of the concatenated ITS, *tef1-α*, *TUB* and *ACT* sequence dataset comprising 1,426 bp revealed 129 parsimony-informative characters. The outgroup taxon was *Botryosphaeriadothidea*. The heuristic search with random addition of taxa (1,000 replicates) generated 5,000 most parsimonious trees of 141 steps (CI = 0.972, RI = 0.990, RC = 0.962, HI = 0.028). In both analyses (MP and ML), *M.phaseolina* and *M.vaccinii* formed a well-supported clade (MP BS = 99%, ML BS = 91%). *Macrophominapseudophaseolina* and *M.euphorbiicola* formed another clade which lacks of bootstrap support (MP BS = 68%, ML BS = 67%, Fig. 1).

### Taxonomy

#### Macrophomina vaccinii

Taxon classificationFungiBotryosphaerialesBotryosphaeriaceae

Y. Zhang ter & L. Zhao sp. nov.

830282

Figure 2

##### Holotype.

CHINA, Fujian province, Nanping city, Jianyang district, Huilong village, from blighted stem of southern high bush (*Vacciniumcorymbosum* × *V.darrowii*), 26 Feb. 2018, L. Zhao (HMAS 255479): ex-type living culture, CGMCC 3.19503.

##### Etymology.

from “*Vaccinium*”, in reference to the host genus.

##### Description.

Sexual stage not observed. Asexual stage: *Sclerotia* developing on SNA, black, smooth, hard, 40–100 µm diam. *Conidiomata* pycnidial, dark brown to black, solitary or gregarious, up to 400 µm diam., each opening by a central ostiole. *Conidiogenous cells* lining the inner surface of the conidioma, hyaline, subcylindrical, each proliferating several times percurrently near the apex, 9–16 × 3–4 µm, young conidiogenous cells each covered by a mucous layer that extends over the apex of the developing conidium. *Conidia* ellipsoid to obovoid, smooth, (18–)20–29(–33) × (8–)9–11(–12) µm (av. 24.8 × 10.1 µm, n = 60, L/W ratio = 2.5, range from 2.3 to 2.8), immature conidia hyaline, enclosed in a mucous sheath, that upon dehiscence encloses the top half of the conidium, transformed into two lateral tentaculiform, apical mucoid appendages (type C; Nag Raj 1993), no pigmented conidia observed after 30 days incubation. *Microconidia* aseptate, hyaline, smooth, guttulate to granular, straight to curved, ellipsoid to subcylindrical to irregular, 5–9(–10) × 3–5 µm.

**Figure 2.**
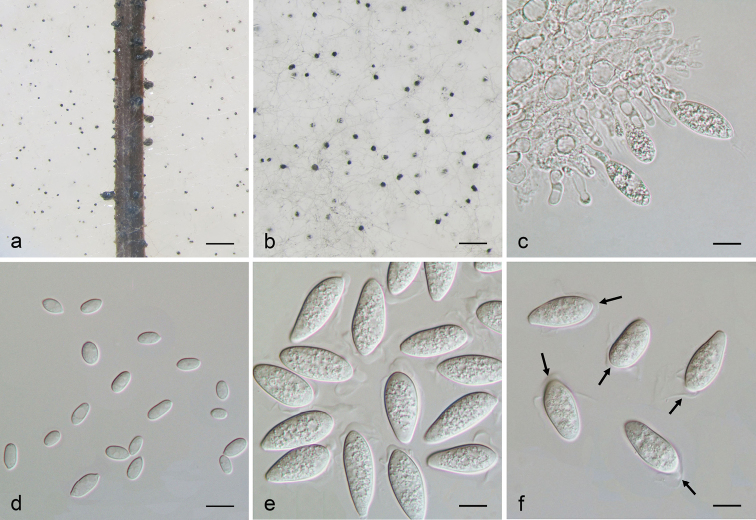
*Macrophominavaccinii* (from ex-type: CGMCC 3.19503). **a** Pycnidia forming on pine needle **b**Sclerotia on the synthetic nutrient-poor agar **c** Conidiogenous cells **d** Microconidia **e–f** Conidia with apical appendages (arrows). Scale bars: 1 mm (**a**); 10 µm (**b–f**).

##### Culture characteristics.

*Colonies* on MEA at 25 °C in darkness, with even margins, sparse aerial mycelia. On MEA buff, turning pale olivaceous to olivaceous-black with dense, black sclerotial masses. Colonies reaching 58.6 mm on MEA after 2 d in the dark at 25 °C.

##### Additional specimens examined.

CHINA, Fujian province, Nanping city, Jianyang district, Huilong village, from blighted stem of southern high bush (*Vacciniumcorymbosum* × *V.darrowii*), 26 February 2018, L. Zhao (Paratype, HMAS 255480): living culture, CGMCC 3.19505; (HMAS 255481): living culture, CGMCC 3.19510.

##### Note.

Based on phylogenetic analysis, *M.vaccinii* and *M.phaseolina* formed a well-supported clade. Morphologically, the wider conidia of *Macrophominavaccinii* can be distinguishable from *M.phaseolina* ((8–)9–11(–12) µm (av. 10.1 µm) *vs.* (6–)8(–9) µm (av. 8 µm)) (Sarr et al. 2014). In addition, the larger-sized pycnidia of *M.vaccinii* are also distinguishable from *M.phaseolina* (up to 400 µm diam. *vs.* up to 300 µm diam.) (Sarr et al. 2014). A comparison of the 264 nucleotides across the *tef1-α* gene region of *M.vaccinii* and *M.phaseolina* (CBS 227.33) reveals 5 base pair differences (1.9%) (Table 3).

### Pathogenicity test

**Figure 3.**
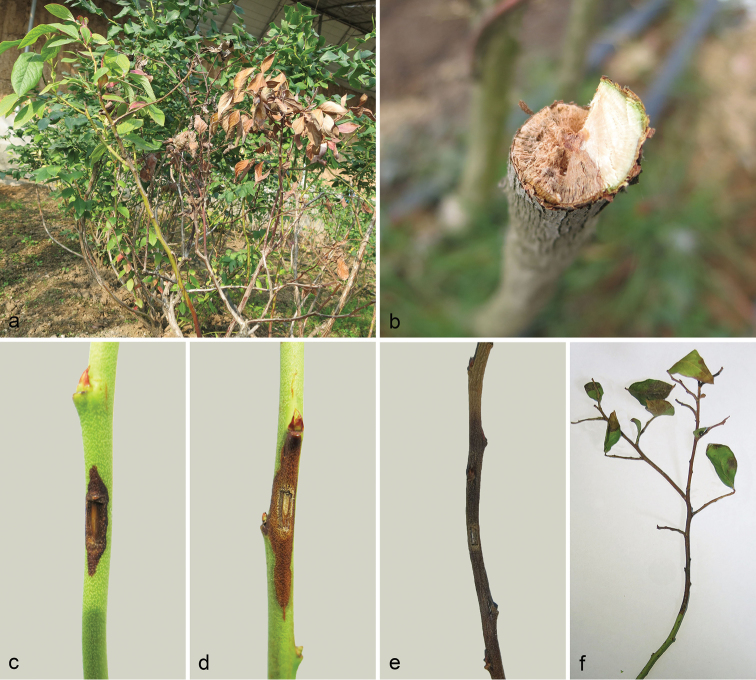
*Macrophominavaccinii* causes stem blight of blueberry. **a** Death of the blueberry (*Vaccinium* spp.) plants in the field **b** Symptoms of stem blight of blueberry in the field **c** Symptoms of *Macrophominavaccinii* after three days inoculation **d** Symptoms of *Macrophominavaccinii* after one-week inoculation **e** Symptoms of *Macrophominavaccinii* after three weeks inoculation **f** Symptoms of blueberry twig of *Macrophominavaccinii* after three weeks inoculation.

All the three isolates of *Macrophominavaccinii* (CGMCC 3.19503, CGMCC 3.19505, and CGMCC 3.19510) were pathogenic on the blueberry stems. Brown lesions appeared on the inoculated spots after 3 days of inoculation for mycelia (Fig. 3). The diseased spots turned brown and lesion area enlarged after 7 days inoculation (Fig. 3). After inoculation for 3 weeks, the length of necrotic lesion reached up to 20 cm, and the infected xylem tissue turned light-brown (Fig. 3). The wounded area of the inoculated stems was the one that was most significantly higher than those of the control groups, while no significant difference was detected among these three inoculated treatments (Fig. 3, Table 2).

Koch’s postulates were performed by successful pathogen re-isolation from all the necrotic stems. The morphology and DNA sequences of these new isolates were consistent with the initial inoculate.

**Table 2.** Pathogenicity on 2-year blueberry stems (*cv.* O’Neal) using mycelia of *Macrophominavaccinii* after 3 weeks.

Species	Isolate	Blueberry stems inoculated with Mycelia ± SD (cm)
* Macrophomina vaccinii *	CGMCC 3.19503	12.63 ± 7.32 a
* Macrophomina vaccinii *	CGMCC 3.19505	12.38 ± 0.48 a
* Macrophomina vaccinii *	CGMCC 3.19510	10.75 ± 2.87 a
Noninoculated control	–	0.00 ± 0.00 b

Note: Data followed by different letters in each column are significantly different based on HSD tests at the P< 0.05 level.

**Table 3.** Major *tef1-a* and *TUB* and *ACT* base pair differences of *Macrophominavaccinii*, *M.phaseolina*, *M.pseudophaseolina* and *M.euphorbiicola*.

Species	Base pair difference	Position of nucleotides difference		
		*tef1-a*	*TUB*	*ACT*
*M.vaccinii* and *M.phaseolina*	G instead of A	11	–	–
	C instead of T	41	–	–
	C instead of G	48	–	–
	A instead of C	75	–	–
	A instead of G	160	–	–
	T instead of C	–	–	76
*M.vaccinii* and *M.pseudophaseolina*	A instead of G	10, 24	–	–
	C instead of T	27, 31, 48, 103, 186	280, 313	–
	G instead of A	101, 144, 208	119, 192	–
	A instead of T	142	–	–
	T instead of C	145, 197, 217, 227, 247	56	76, 192
	T instead of A	219	–	–
	C instead of A	–	202	–
*M.vaccinii* and *M.euphorbiicola*	C instead of T	14, 23, 33, 193, 221	280, 313	–
	A instead of G	24	–	–
	T instead of C	43, 250	56	76, 192
	C instead of G	48	–	–
	C instead of A	106	202	–
	G instead of A	144, 211	119, 192	83
	A instead of C	185	–	–
	G instead of C	–	200	–

## Discussion

*Macrophomina* is a cosmopolitan genus, with a broad host range and colonizing more than 500 crops and non-crop species, such as soybean, common bean, corn, sorghum, cowpea, peanut and cotton (Su et al. 2001, Ndiaye et al. 2010, Sarr et al. 2014, Sun et al. 2015). In this study, *Macrophominavaccinii* was collected from the lesion of stem blight in Fujian province in China, a subtropical area in China. *Macrophominaphaseolina*, the most common species of *Macrophomina*, is considered as economically more important in subtropical and tropical countries with semi-arid climates, which tends to occur in hot and dry conditions (Wrather et al. 1997, 2001, Smith and Wyllie 1999, Radwan et al. 2014). Charcoal rot of beans is caused by *M.phaseolina*, however, this has frequently been reported in the northern part of China, with a disease incidence of 80% in Beijing and Tianjin (Zhang et al. 2009, 2011, Sun et al. 2015).

So far, seven species have been assigned within *Macrophomina*, *viz. M.euphorbiicola*, *M.limbalis*, *M.phaseoli*, *M.phaseolina*, *M.philippinensis*, *M.pseudeverniae* and *M.pseudophaseolina*. However, *M.limbalis* was transferred to *Dothiorella* (as *D.limbalis*), *M.pseudeverniae* to *Didymocyrtis* (as *D.pseudeverniae*), while *M.phaseoli* and *M.philippinensis* were treated as the synonym of *M.phaseolina*. Thus, only three species, *viz. M.euphorbiicola*, *M.phaseolina* and *M.pseudophaseolina* are currently accommodated within *Macrophomina*. Morphologically, wider conidia of *M.vaccinii* ((8–)9–11(–12) µm) are distinguishable from *M.phaseolina* ((6–)8(–9) µm) and *M.pseudophaseolina* ((7.5–)8(–9) µm) (Sarr et al. 2014). The larger-sized pycnidia of *M.vaccinii* (up to 400 µm diam.) can also be distinguishable from *M.phaseolina* (up to 300 µm diam.) and *M.pseudophaseolina* (up to 300 µm diam.) (Sarr et al. 2014). In addition, the smaller-sized sclerotia of *M.vaccinii* (40–100 µm diam.) also differs from *M.phaseolina* (100–400 µm diam.) and *M.pseudophaseolina* (100–400 µm diam.) (Sarr et al. 2014). *Macrophominaeuphorbiicola* lacks morphological descriptions, and only DNA sequences are available for species comparison (Machado et al. 2019).

Phylogeny based on concatenated ITS, *tef1-α*, *TUB* and *ACT* DNA sequences indicated that the subclade comprising eight isolates of *Macrophominavaccinii* are closely related to *M.phaseolina* (Fig. 1). A comparison of the *tef1-α* regions DNA sequence data of *M.vaccinii* and *M.phaseolina* revealed a 1.9% base pair difference. A comparison of the 266 nucleotides across the *tef1-α* gene region of *M.vaccinii* and *M.pseudophaseolina* (CPC 21417) reveals 17 base pair differences (6.39%). Although the morphological characteristics of *M.euphorbiicola* cannot be obtained, a comparison of the 269 nucleotides across the *tef1-α* gene region between *M.vaccinii* and *M.euphorbiicola* (CMM 4134) shows 13 base pair differences (4.83%) (Table 3). Following the recommendations of Jeewon and Hyde (2016) and Tennakoon et al. (2018), there is sufficient evidence to justify our taxon as a new species.

Pathogenicity tests conducted on 2-year blueberry stems (*cv.* O’Neal) indicated that inoculation of *Macrophominavaccinii* were pathogenic on blueberry stems which causes the stem turn brown with necrotic lesions. Similar symptoms caused by *M.phaseolina* have been reported on blueberry in Serbia, resulting in foliage death, and brown discoloration of internal vascular tissues at the basal part of the bush (Popović et al. 2018). The brown lesion caused by *M.vaccinii* and *M.phaseolina* on blueberries differs from the widely reported charcoal rot diseases caused by *Macrophominaphaseolina* and *M.pseudophaseolina* (Su et al. 2001, Salik 2007, Yang et al. 2005, Zhang et al. 2011, Sarr et al. 2014, Sun et al. 2015).

## Supplementary Material

XML Treatment for Macrophomina vaccinii
